# An evaluation of the relationship between BIS and MOAA/S during remimazolam-sufentanil induction: a prospective observational study

**DOI:** 10.3389/fmed.2026.1877209

**Published:** 2026-09-04

**Authors:** Jun-li Zheng, Yi Cai, Yun-xia Zhang, Jian Shen, Xiao-dong Huang, Wei-long Wang, Zhen-feng Zhou

**Affiliations:** 1 Department of Anesthesiology, The First People’s Hospital of Lin-ping District, Hangzhou, China; 2 The Fourth Clinical Medical College, Zhejiang Chinese Medicine University, Hangzhou, China; 3 Department of Anesthesiology, Hangzhou Women’s Hospital (Hangzhou Maternity and Child Health Care Hospital), Hangzhou, China; 4 Department of Anesthesiology, Affiliated Hangzhou First People’s Hospital, School of Medicine, Westlake University (The Forth Clinical Medical College, Zhejiang Chinese Medicine University), Hangzhou, China

**Keywords:** agreement, BIS, correlation, MOAA/S, remimazolam

## Abstract

**Background:**

Both the Bispectral Index (BIS) and the Modified Observer’s Assessment of Alertness/Sedation (MOAA/S) scale are commonly used to evaluate the depth of anesthesia in studies investigating the effective dosing of remimazolam. However, their correlation remains unclear. This prospective observational study aimed to characterize the correlation between BIS values and MOAA/S scores during induction of anesthesia with remimazolam–sufentanil in elderly and non-elderly patients.

**Methods:**

Sixty patients were enrolled and stratified into elderly and non-elderly cohorts (*n* = 30 each). All participants received intravenous sufentanil (0.1 μg/kg), followed by remimazolam infusion (0.16 mg/kg in elderly patients; 0.22 mg/kg in non-elderly patients). BIS values and corresponding MOAA/S scores were recorded at 1-min intervals for 5 min after induction. The minimum BIS value and its corresponding MOAA/S score were also captured. Spearman’s rank correlation and Cohen’s kappa coefficient were used to assess the relationship and agreement between BIS < 60 and MOAA/S = 0. A generalized linear mixed model (GLMM) was applied to evaluate the association between BIS and the probability of deep sedation (MOAA/S = 0).

**Results:**

The correlation between BIS and MOAA/S showed a dynamic pattern, demonstrating a moderate association at the initiation of induction (T1; *r* = 0.681, *p* < 0.001) that progressively weakened over time. The area under the receiver operating characteristic curve (AUC) of BIS for predicting deep sedation (MOAA/S = 0) was 0.635 in elderly patients and 0.780 in non-elderly patients. The GLMM identified a significant non-linear association between BIS and the probability of deep sedation (χ^2^ (1) = 6.62, *p* = 0.010). Overall agreement between BIS and MOAA/S was slight (κappa = 0.175).

**Conclusion:**

Bispectral Index and MOAA/S exhibit a complex non-linear relationship and slight concordance during induction with remimazolam–sufentanil.

## Introduction

Remimazolam, an ultra-short-acting benzodiazepine, induces sedation through specific GABAergic modulation at central GABAA receptor sites (1). It exhibits a quick onset, rapid elimination, a negligible impact on cardiorespiratory function, and clearance that is not dependent on major organ function. Moreover, its effects could be promptly reversed with flumazenil (2–4). Owing to these favorable pharmacokinetic and pharmacodynamic properties (5), remimazolam had gained increasing clinical use in anesthetic practice.

Accurate monitoring of sedation depth during general anesthesia was crucial for optimizing anesthetic delivery, preventing intraoperative awareness, and improving overall clinical outcomes (6). Two commonly used monitoring modalities were the Bispectral Index (BIS)-an electroencephalogram-derived measure of cortical activity-and the Modified Observer’s Assessment of Alertness/Sedation (MOAA/S) scale, a clinical tool based on patient responsiveness. The BIS and the MOAA/S scale are extensively utilized in research investigating the dosage efficacy and sedative profile of remimazolam (7–11). While MOAA/S was often regarded as the reference standard for behavioral assessment of sedation during remimazolam administration (10, 11). However, BIS is considered to be an objective indicator that can reliably monitor the sedation depth of remimazolam based on emerging evidence (8, 9). Previous studies have reported strong correlations between BIS values and MOAA/S scores when using sedatives such as propofol and dexmedetomidine (12, 13).

However, the correlation between BIS values and MOAA/S scores during remimazolam-induced anesthesia-particularly during the dynamic induction phase-remains insufficiently defined. To address this gap, we conducted a prospective observational study to evaluate the correlation between BIS and MOAA/S during induction with remimazolam-sufentanil in both elderly and non-elderly patients.

## Materials and methods

### Study design

This study protocol received ethical approval (IRB: No. 2025-009) on November 04, 2025. Written informed consent was obtained from all participants beforehand. Subsequently, 65 patients under general anesthesia were enrolled from November 08 to December 05, 2025.

### Inclusion and exclusion criteria

Eligible patients met all of the following criteria: they (1) aged between 18 and 80 years; (2) had an American Society of Anesthesiologists (ASA) physical status classification of I or II; (3) were scheduled for elective surgery with general anesthesia;(4) had a body mass index (BMI) between 18 and 30 kg/m^2^; and (5) were able to voluntarily give informed consent in writing.

While patients met the following exclusion criteria were included: (1) non-elective surgery; (2) known any contraindication to remimazolam or to its use; (3) pre-existing cognitive impairment, chronic use of analgesics (e.g., opioids, NSAIDs) or psychotropic medications (e.g., sedatives, antidepressants), or a history of alcohol abuse; (4) Sedatives, antiemetic drugs and antipruritic drugs were used within 24 h before surgery as well as antidepressants or monoamine oxidase inhibitors were used within 15 days; (5) anticipated difficult airway management; (6) significant hepatic or renal dysfunction, gastrointestinal ulceration, or coagulopathy; (7) cases in which the monitored EEG signal was of suboptimal quality or incomplete. (8) Declined to participate or current enrollment in another interventional clinical trial.

### Blinding and drug preparation

Based on age, the patients were divided into two groups: Group O (elderly, aged 65–80 years, *n* = 30) and Group N (non-elderly, aged 18–64 years, *n* = 30). An independent nurse, not involved in clinical management or data collection, prepared the study drug of remimazolam. The effective induction dose of remimazolam for general anesthesia varies with age; in accordance with our prior dose-response study (14), elderly and non-elderly patients received remimazolam (Lot Number: H20190034, Jiangsu Hengrui Pharmaceuticals Co., Ltd.) at 0.16 mg/kg and 0.22 mg/kg, respectively. Remimazolam was reconstituted in 0.9% normal saline to a total volume of 20 mL according to the patient’s body weight. The attending anesthesiologist, who was blinded to the prepared remimazolam concentration and dose, performed all anesthetic procedures, including drug administration. The same anesthesiologist assessed the Modified Observer’s Assessment of Alertness/Sedation (MOAA/S) score and reported the results to an independent data collector. All anesthesiologists responsible for MOAA/S assessments received standardized training before study initiation. BIS was assessed by a separate independent investigator who was blinded to the MOAA/S results. The independent data collector and data analysts were all blinded to the group assignments and medication details throughout the trial.

### Anesthesia procedure

Patients received no preoperative sedatives. Upon arrival in the operating room, standard monitoring (HR, NIBP, SpO2) was commenced and was supplemented with invasive monitoring as clinically required. Prior to sensor placement, and in accordance with the manufacturer’s guidelines, the skin was cleansed with alcohol wipes for degreasing and to promote adhesion before positioning the disposable BIS Quatro sensor (Medtronic, Model 186-0106). BIS was monitored continuously throughout the procedure. Only BIS recordings with a signal quality index (SQI) ≥ 80% were retained for analysis. Data affected by high electrode impedance (>5 kΩ), electrosurgical interference, or patient movement were excluded.

Pre-oxygenation was performed via a face mask before the induction of anesthesia. An initial intravenous dose of 0.1 μg/kg sufentanil (Lot Number: H20054171, Yichang Renfu Pharmaceutical Co., Ltd.) was administered. After an interval of 1 min, remimazolam was administered intravenously at 0.16 mg/kg for elderly patients or 0.22 mg/kg (14) for non-elderly patients over 30 s using a syringe pump. After drug administration, BIS values and MOAA/S scores were recorded sequentially at 1-min intervals for 5 min. Each minute, the BIS value was documented first, followed immediately by MOAA/S assessment using a standardized stepwise stimulus protocol: verbal call, light shaking, and trapezius squeeze, progressing only in the absence of a response.

Five minutes post-remimazolam injection, if the MOAA/S score was ≥1, a rescue infusion of propofol (Lot Number: H19990282, Xi’an Libang Pharmaceutical Co., Ltd.) (1 mg/kg) was given and repeatable every 3 min until MOAA/S = 0. Upon achieving adequate sedation, supplemental 0.5 μg/kg sufentanil and 0.8 mg/kg rocuronium (Lot Number: H20183305, Emei Mountain Tonghui Pharmaceutical Co., Ltd.) were administered. Manual ventilation was then initiated, followed by video laryngoscope-assisted tracheal intubation 3 min later. After confirming correct tube placement and fixation, the patient was connected to the anesthesia machine for mechanical ventilation. Anesthesia maintenance was conducted with remimazolam, sufentanil, rocuronium, and sevoflurane (Lot Number: H20213735, Shanghai Hengrui Pharmaceutical Co., Ltd.).

### Define of successful sedation

Sedation was considered successful if an MOAA/S score of 0 (15) was achieved within 5 min after remimazolam administration.

### Outcome measures

Sedation effectiveness was evaluated using either BIS monitoring (16) (a BIS value below 60) or MOAA/S assessment (15) (an MOAA/S score of 0) within 5 min after remimazolam administration. BIS values and MOAA/S scores were recorded at 1-min intervals during the first 5 min after induction with remimazolam. The minimum BIS value observed and its corresponding MOAA/S score were also documented (The MOAA/S evaluation criteria are provided in Supplementary Table 1).

Other outcomes included:

Respiratory depression (5): Respiratory rate lower than 8 breaths/min over 1 min or SpO_2_ lower than 90%; managed with jaw thrust maneuver and increase oxygen supplementation.

Hypotension (17): Systolic blood pressure lower than 90 mmHg or a reduction ≥30% from baseline; managed with 0.5 mg intravenous metaraminol.

Bradycardia (18): Heart rate lower than 50 bpm over 30 s; managed with 0.5 mg intravenous atropine.

Hiccups: it would be recorded if suffered.

Data were obtained at baseline (pre-injection) and at 1-min intervals during the initial 5-min period post-induction (T1–T5) to ensure comprehensive monitoring.

### Sample size

An *a priori* sample size calculation was performed with G*Power software (version 3.1). Assuming a two-tailed test with an α level of 0.05 and a power of 80%, the primary objective was to detect a correlation between BIS values and MOAA/S scores during remimazolam-induced anesthesia. Based on data from a pilot study (14) and prior literature examining associations between sedation monitors and clinical scales (19), we anticipated a moderate Spearman’s correlation coefficient (ρ = 0.5). Under these assumptions, a minimum of 29 participants per group (elderly and non-elderly) was required. Ultimately, 60 patients were enrolled, with 30 participants allocated to each group.

### Statistical analysis

All statistical analyses were conducted using SPSS (version 25.0; IBM Corp.) and R (version 4.5.1; R Foundation). Based on their distribution, continuous variables were expressed as mean ± standard deviation (SD) or as median (interquartile range, IQR). Categorical variables are presented as number (percentage).

Spearman’s correlation was performed to assess the association between MOAA/S scores and BIS values. The discriminative ability of BIS to identify deep sedation (MOAA/S = 0) was evaluated via receiver operating characteristic (ROC) curve analysis. For the ROC analysis, only the minimum BIS value recorded for each patient was included. This value was selected to represent the peak hypnotic effect and to ensure that each patient contributed a single observation, thereby maintaining statistical independence.

A generalized linear mixed-effects model (GLMM) was applied to characterize the non-linear association between BIS and successful sedation (MOAA/S = 0). BIS was modeled as a fixed effect using a natural spline with two degrees of freedom, with age and sex included as additional fixed-effect covariates. A patient-specific random intercept accounted for repeated measurements. A binomial distribution with a logit link was used, and a likelihood ratio test assessed the significance of the non-linear spline term.

Agreement between “BIS < 60” and “MOAA/S = 0” was evaluated using Cohen’s kappa coefficient. According to Landis and Koch (20), kappa values were interpreted as: <0 (no agreement), 0.00–0.20 (slight agreement), 0.21–0.40 (fair agreement), 0.41–0.60 (moderate agreement), 0.61–0.80 (substantial agreement), and 0.81–1.00 (almost perfect agreement). For all tests, a two-sided *p* < 0.05 was considered as statistically significance.

## Results

### Baseline characteristics

**FIGURE 1:**
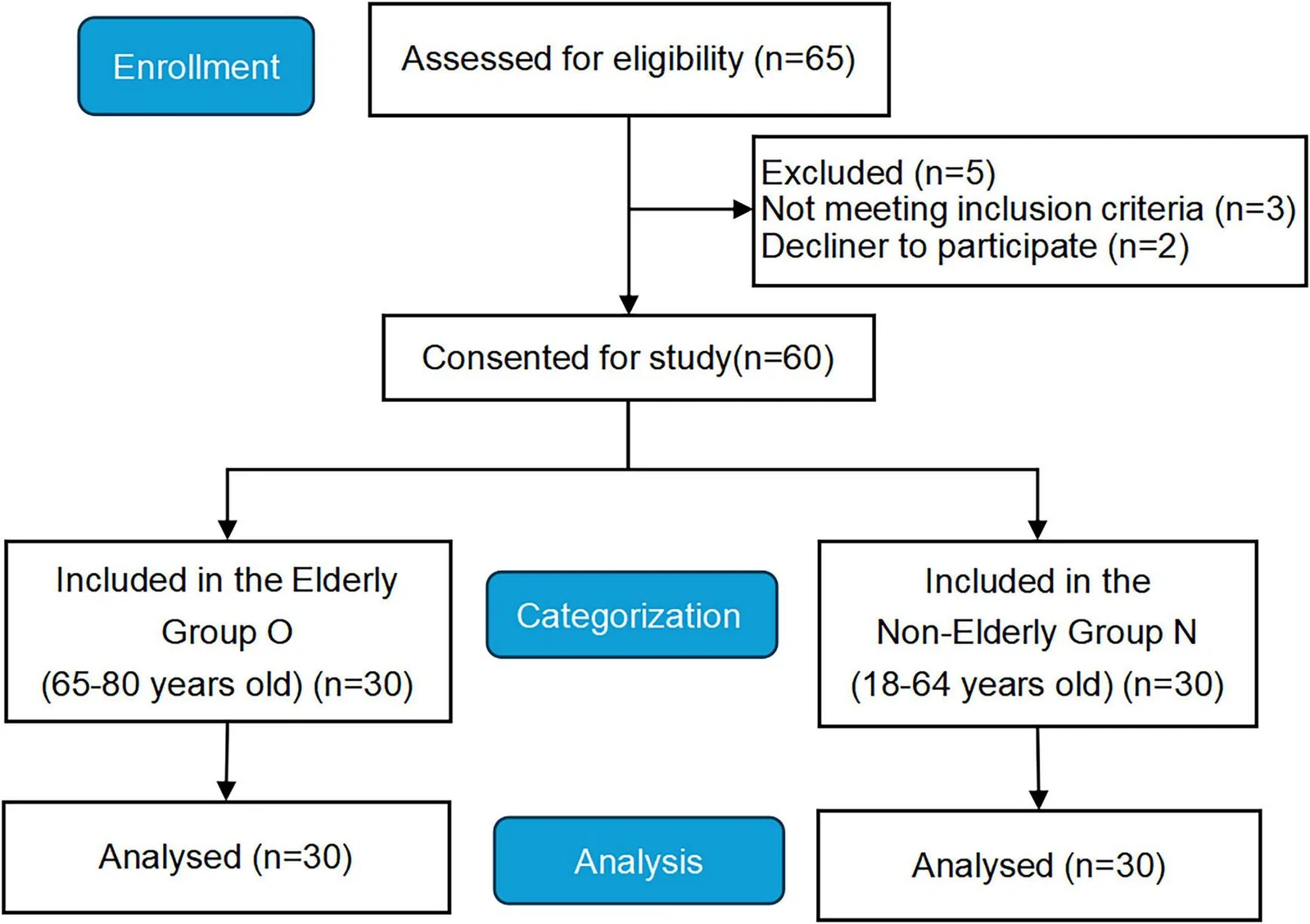
Flow diagram of the study cohort. A total of 65 patients were assessed for eligibility. Five patients were excluded (3 did not meet inclusion criteria, 2 declined participation). The remaining 60 patients were enrolled and categorized into two age-defined cohorts: elderly (≥65 years, *n* = 30) and non-elderly (<65 years, *n* = 30). All enrolled patients were included in the final analysis.

Of the 65 patients screened, 5 were excluded for not meeting the inclusion criteria. The remaining 60 patients were enrolled and included in the final analysis (Figure 1). Baseline demographic and clinical characteristics are summarized in Table 1.

**TABLE 1:** Baseline demographic and clinical characteristics.

	Total	Group O	Group N	*P*-value
Age, y	58.5 ± 14.35	70.0 ± 4.87	46.9 ± 10.92	<0.001
Height, cm	162.3 ± 7.33	162.7 ± 7.45	161.9 ± 7.37	0.999
Weight, kg	60.3 ± 9.69	60.1 ± 8.85	60.5 ± 10.60	0.809
BMI, kg⋅m^–2^	22.8 ± 2.57	22.6 ± 2.45	22.9 ± 2.71	0.990
Gender, *n*				0.434
Male	31	18	13	
Female	29	12	17	
ASA, *n*				0.206
I	3	0	3	
II	57	30	27	
Coexistent disease, n (%)				
Hypertension	33 (55)	24 (80)	9 (30)	0.001
Diabetes	5 (8.3)	3 (10)	2 (6.6)	0.897
COPD	1 (1.7)	1 (3.3)	0 (0)	0.601
BIS	96.2 ± 2.07	96.4 ± 2.21	95.9 ± 1.93	0.721

BMI, body mass index; ASA, American Society of Anesthesiologists; COPD, chronic obstructive pulmonary disease; BIS, Bispectral Index.

### Correlation of BIS and MOAA/S scores

**FIGURE 2:**
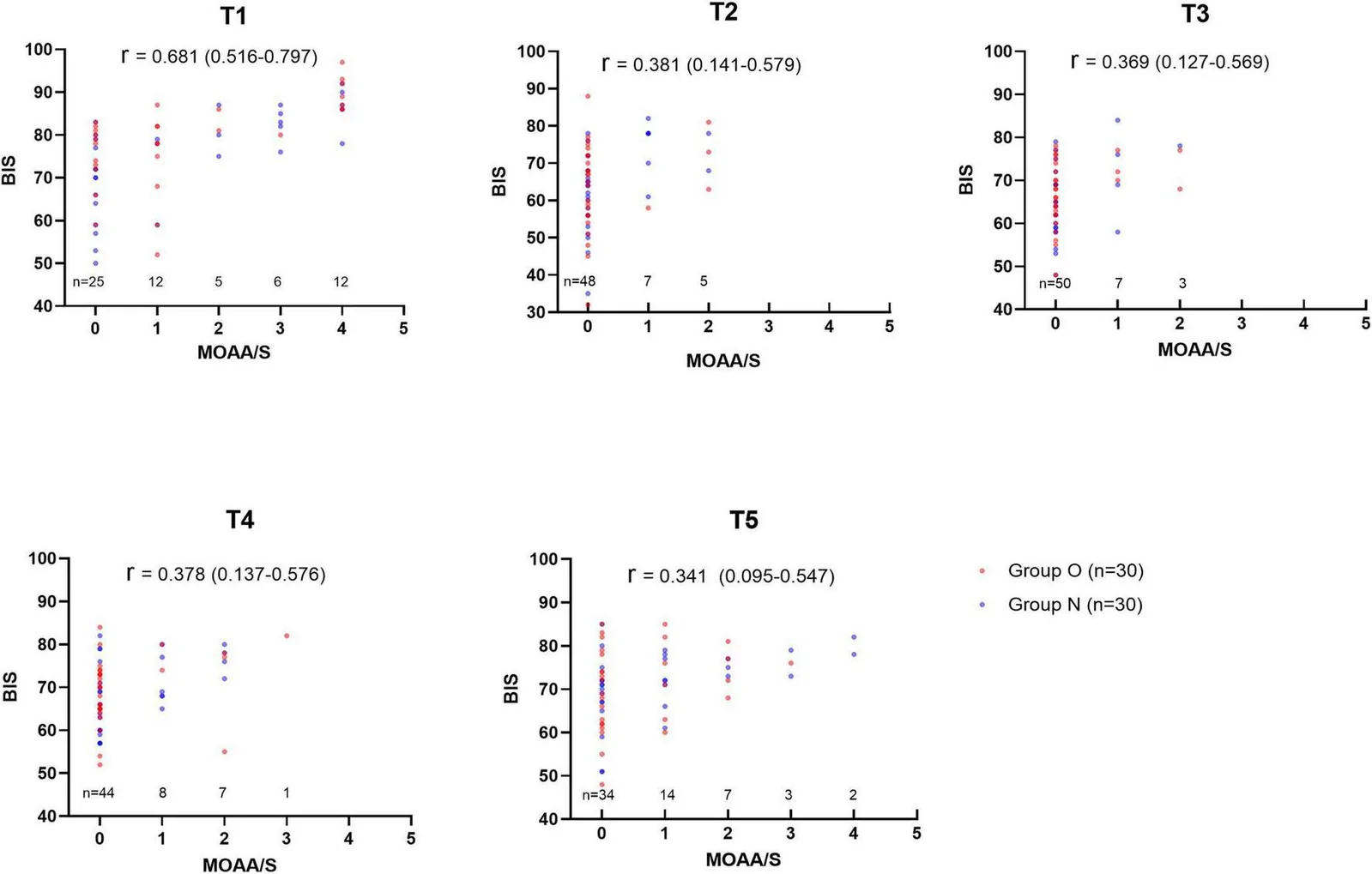
Scattergram of correlation between BIS and MOAA/S scale at each time point. The *r*-values represent Spearman’s correlation coefficient.

Figure 2 illustrates scatter plots demonstrating the correlation between BIS values and MOAA/S scores at each measured time point. At T1, Spearman correlation analysis revealed a statistically significant moderate positive correlation (*r* = 0.681, 95% CI: 0.516–0.797, *p* < 0.001). However, correlations at subsequent time points (T2–T5) were weak, with coefficients of 0.381 (95% CI: 0.141–0.579, *p* = 0.003), 0.369 (95% CI: 0.127–0.569, *p* = 0.004), 0.378 (95% CI: 0.137–0.576, *p* = 0.003), and 0.341 (95% CI: 0.095–0.547, *p* = 0.008), respectively.

**FIGURE 3:**
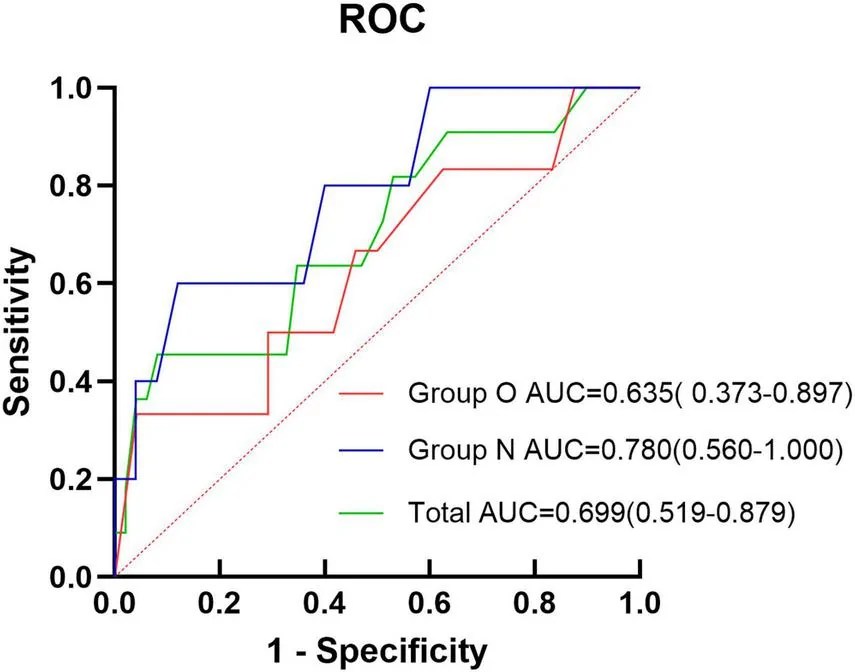
Receiver operating characteristic (ROC) curves for different patient groups. The AUC was highest in the non-elderly group, followed by the total patient cohort, and was lowest in the elderly group.

The ROC analysis (Figure 3) showed that BIS had limited discriminative ability for predicting MOAA/S = 0. The area under the curve (AUC) was 0.635 (95% CI: 0.373–0.897) in elderly patients, 0.780 (95% CI: 0.559–1.000) in non-elderly patients, and 0.699 (95% CI: 0.519–0.879) in the overall cohort.

**FIGURE 4:**
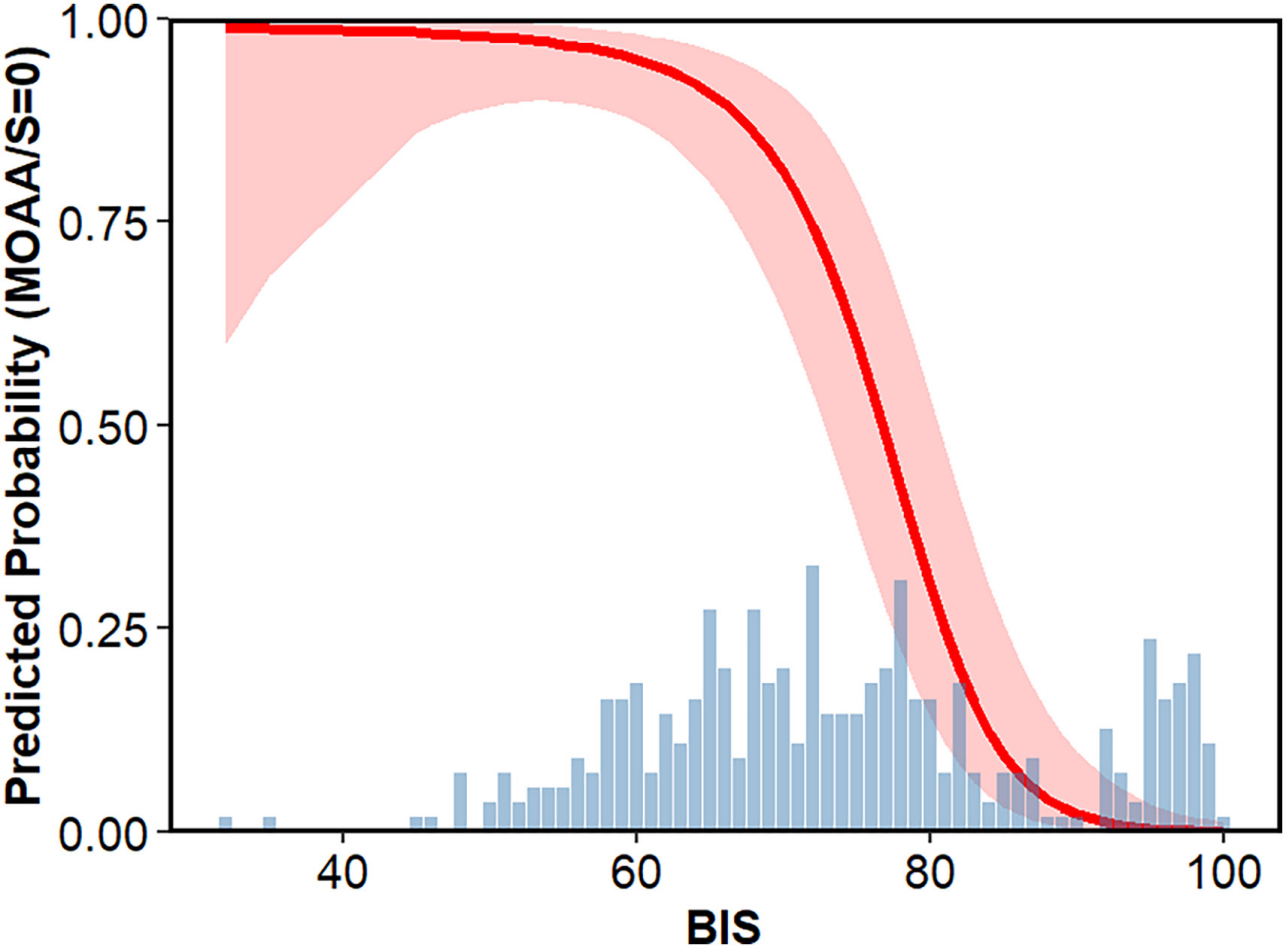
The generalized linear mixed model. Likelihood ratio test: χ^2^ (1) = 6.62, *p* = 0.010.

After adjustment for age, sex, and patient-level variability, the generalized linear mixed-effects model (GLMM) demonstrated a statistically significant non-linear association between BIS and the probability of MOAA/S = 0 (likelihood ratio test: Δdf = 1, χ^2^ = 6.62, *p* = 0.010) (Table 2, Figure 4).

**TABLE 2:** GLMM comparison and regression coefficient summary for BIS spline terms with different degrees of freedom.

	AIC	Estimate	SE	z	*P*	95% CI
df = 1	281.724					
ns (BIS, df = 1)		−18.292	2.24	−8.166	<0.001	−22.682 to −13.902
df = 2	277.105					
ns (BIS, df = 2)1		−8.976	3.575	−2.511	0.012	−15.984 to −1.969
ns (BIS, df = 2)2		−11.077	1.446	−7.659	<0.001	−13.911 to −8.242
df = 3	278.406					
ns (BIS, df = 3)1		−5.49	1.706	−3.219	0.001	−8.833 to −2.147
ns (BIS, df = 3)2		−13.635	6.848	−1.991	0.046	−27.057 to −0.212
ns (BIS, df = 3)3		−12.515	2.433	−5.144	<0.001	−17.283 to −7.747
df = 4	279.811					
ns (BIS, df = 4)1		−4.588	3.532	−1.299	0.194	−11.511 to 2.335
ns (BIS, df = 4)2		−5.836	2.613	−2.233	0.026	−10.958 to −0.714
ns (BIS, df = 4)3		−12.439	7.922	−1.57	0.116	−27.966 to 3.088
ns (BIS, df = 4)4		−13.46	3.005	−4.479	<0.001	−19.351 to −7.57

There were 23.3% of elderly patients and 30% of non-elderly patients reached a MOAA/S score of 0 (deep sedation), while their BIS values remained ≥60 (Supplementary Figure 1).

### Agreement between BIS < 60 and MOAA/S = 0

Cohen’s kappa analysis revealed low levels of agreement between BIS and MOAA/S across all analyses. The non-elderly, elderly, and overall cohorts exhibited Kappa coefficients of 0.255 (95% CI: 0.155–0.364, *p* < 0.001), 0.102 (95% CI: 0.018–0.186, *p* = 0.017), and 0.175 (95% CI: 0.093–0.261, *p* < 0.001), respectively, consistent with slight to fair agreement between the two assessment modalities (Table 3).

**TABLE 3:** Agreement between BIS and MOAA/S scores.

MOAA/S	BIS		Kappa-value	*P*-value
	Effective	Ineffective		
Group O			0.102	0.017
Effective	18	87		
Ineffective	4	71		
Group N			0.255	<0.001
Effective	28	68		
Ineffective	2	82		
The total			0.175	<0.001
Effective	46	155		
Ineffective	6	153		

It is valid when MOAA/S = 0 and/or BIS < 60. When kappa coefficients <0 indicate no agreement; 0.0–0.2, slight agreement; 0.21–0.4, fair agreement; 0.41–0.6, moderate agreement; 0.61–0.8, substantial agreement; and 0.81–1.0, almost perfect agreement.

### Adverse events

No significant intergroup differences were observed in the incidence of hiccups, bradycardia, hypotension, or respiratory depression within 5 min after remimazolam induction (all *p* > 0.05; Supplementary Table 2).

## Discussion

In this study, BIS demonstrated slight agreement with MOAA/S scores during induction of anesthesia with remimazolam–sufentanil (overall κappa = 0.175). This finding contrasts with prior studies involving propofol and dexmedetomidine, which reported strong correlations between BIS and MOAA/S scores (12, 13). Notably, 23.3% of elderly patients and 30% of non-elderly patients reached a MOAA/S score of 0 (deep sedation), while their BIS values remained ≥60. Therefore, multidimensional monitoring that combines clinical assessment with objective electroencephalographic indices may be warranted to provide a more comprehensive assessment of anesthetic depth during remimazolam-based anesthesia.

Our findings are consistent with those reported by Yu-Hang Cai et al. (21). In their study, following the intravenous administration of remimazolam in pediatric patients, the BIS value showed a poor correlation with the MOAA/S score. They observed that BIS values seldom dropped below 60 even at MOAA/S scores below 1. Clinically, the observed non-linear relationship between BIS and MOAA/S scores indicated that a fixed reduction in the BIS value does not uniformly correspond to a consistent change in sedation depth. For example, during the progression from moderate to deep sedation (MOAA/S 3-0) (22), the decline in BIS becomes steep, followed by a plateau phase at MOAA/S scores of 0, where BIS may decrease only slightly or remain unchanged (23). This phenomenon highlights the complexity of interpreting BIS across different sedation stages.

Notably, the area under the curve (AUC) was 0.635 in elderly patients, 0.780 in non-elderly patients. Considering that the opioid component of our anesthetic regimen was not expected to substantially influence BIS, while the benzodiazepine was anticipated to exert only a moderate effect, the reliability of BIS readings in non-elderly patients appears particularly encouraging. Opioids are known to enhance MOAA/S sensitivity by inhibiting spinal reflexes, while their effect on BIS is comparatively limited (24, 25). The observed differences in BIS performance between the two age cohorts suggest that BIS values should be interpreted with caution in older patients, whereas its predictive performance appeared more reliable in the non-elderly population.

Bispectral Index is a multivariable EEG-derived index of cortical activity (26), whereas the MOAA/S score is a behavioral measure of responsiveness to external stimuli (27) and may be influenced by delayed motor responses (28). Under deep sedation, cortical activity may be profoundly suppressed, resulting in a low BIS value, while the spinal cord and brainstem may retain the capacity to generate motor responses to intense noxious stimulation (22, 29, 30). Thus, although BIS may accurately reflect cortical suppression, it may not reliably predict the occurrence of movement in response to surgical stimulation. However, MOAA/S assessment stimulation during one assessment might still influence BIS measurement at subsequent time points.

Pharmacologically, remimazolam acts selectively on GABAA receptors (2). Once receptor saturation is achieved, further increases in remimazolam concentration result in minimal additional EEG suppression. Similar dissociation has been reported with midazolam, wherein BIS values rarely fall below 60 despite profound sedation (31, 32). In contrast, agents such as propofol and inhalational anesthetics exert their effects across multiple molecular targets (31, 33). Studies on dexmedetomidine and propofol have demonstrated comparable performance of BIS and MOAA/S scores in monitoring sedation depth (12, 13). However, BIS may underestimate sedation depth during benzodiazepine-induced hypnosis due to a weak correlation between γ-oscillation suppression and the BIS algorithm (34), particularly when co-administered with opioids. Sufentanil–a potent opioid analgesic–can exert sedative effects by inducing cortical EEG patterns resembling anesthesia or sleep, thereby reducing arousal (35). Thus, the accuracy of BIS monitoring under remimazolam anesthesia remains debatable. In contrast to the prevalent use of the MOAA/S scale for assessing LOC, the application of BIS in remimazolam studies is considerably less common.

Some studies, non-etheless, have supported the utility of BIS in monitoring sedation with benzodiazepines. For example, studies involving midazolam and propofol in both young and elderly populations reported strong correlations between BIS and Ramsay Sedation Scale (RSS) scores (36, 37). Furthermore, recent studies involving remimazolam demonstrated acceptable correlation between BIS and the Patient State Index (PSI), suggesting that BIS may be a reliable hypnotic monitor (8, 9). However, those studies differed from ours in key methodological aspects: they did not define “successful sedation” and relied primarily on Pearson correlation coefficients rather than consistency measures such as Kappa statistics. In our study, sedation efficacy was explicitly defined using BIS < 60, and consistency was evaluated via Kappa analysis. This methodological distinction further emphasizes that BIS alone may not reliably reflect behavioral sedation depth during remimazolam-based anesthesia.

Rather than the ED95 established in our prior work (14), the remimazolam induction dose was set at the ED50 to optimize the evaluation of anesthetic depth monitoring. At the ED95 level (defined by BIS < 60), subjects exhibited minimal variability in MOAA/S scores (predominantly 0–1), effectively creating a ceiling effect for deep sedation. This limited the ability to acquire data for lighter sedation levels (MOAA/S 2–5). Therefore, the ED50 was selected to ensure a wider dispersion of MOAA/S scores. This approach allowed for the successful acquisition of data across the entire sedation scale, which is essential for a valid and reliable analysis of the relationship between BIS values and clinical sedation scores.

Taken together, these findings indicate that reliance on a single monitoring modality-whether EEG-derived or behaviorally based-may be inadequate during remimazolam-induced anesthesia. Where EEG-based monitors are unavailable, MOAA/S scoring remains a practical alternative, but rigorous standardization and training are essential to minimize observer-related variability. Our results support a multi-dimensional approach to monitoring sedation depth during remimazolam anesthesia, integrating BIS with other EEG-derived parameters (e.g., analgesia or nociception indices) and structured behavioral assessments. No single parameter can reliably capture all dimensions of anesthetic depth. In current clinical practice, anesthesiologists should incorporate raw EEG or spectrographic analyses (e.g., dynamic spectral array) into monitoring protocols, with particular attention to identifying specific EEG signatures–such as dominant frequency shifts associated with loss of responsiveness–especially in older patients and those receiving opioid–benzodiazepine combinations, as these insights offer meaningful guidance for real-time clinical decision-making.

### Limitations

We acknowledge several limitations in this study. Firstly, the generalizability of the findings may be constrained by its single-center design. Although the dataset consisted of 360 paired observations, enabling robust detection of correlation and agreement metrics, a larger sample size would further strengthen external validity, particularly in light of interindividual variability. Second, we recognize that the 60-s sampling interval limited our ability to capture rapid transitions. Due to the fast pharmacokinetic profile of remimazolam, subtle yet significant pharmacodynamic changes may have been missed. This temporal limitation might obscure the true correlation between BIS and MOAA/S. Future studies utilizing continuous, high-resolution monitoring are necessary to map the full dynamics of this relationship. Third, our study focused exclusively on the induction phase. Surgical procedures, anesthesia maintenance, and recovery were not controlled for or evaluated. However, MOAA/S evaluation could not be performed when muscle relaxant drugs were used during general anesthesia. Fourth, the subgroup-specific AUC estimates were based on only 30 participants per cohort and may therefore be imprecise. Moreover, the study may not have been adequately powered to detect statistically significant differences in predictive performance between the two age cohorts.

## Conclusion

This study demonstrated a complex, non-linear relationship and slight concordance between BIS and MOAA/S scores during remimazolam-sufentanil induction.

## Data Availability

The original contributions presented in this study are included in this article/Supplementary material, further inquiries can be directed to the corresponding author.
