# Supplementary material for: An evaluation of the relationship between BIS and MOAA/S during remimazolam-sufentanil induction: a prospective observational study
Source: Front Med (Lausanne). 2026 Sep 4;13:1877209. doi: 10.3389/fmed.2026.1877209 (PMC13585711; doi:10.3389/fmed.2026.1877209)
Supplement: Supplementary file 1 [file Supplementary_file_1.doc]

S1 Supplemental Table 1: Responsiveness scores of the modified Observer’s Assessment of Alertness/Sedation scale.

| Scores |  |
| --- | --- |
| 5 | Responds readily to name spoken in normal tone |
| 4 | Lethargic response to name spoken in normal tone |
| 3 | Responds only after name is called loudly or repeatedly |
| 2 | Responds only after mild prodding or shaking |
| 1 | Does not respond to mild prodding or shaking |
| 0 | Does not respond to noxious stimulus |
