# Supplementary material for: An evaluation of the relationship between BIS and MOAA/S during remimazolam-sufentanil induction: a prospective observational study
Source: Front Med (Lausanne). 2026 Sep 4;13:1877209. doi: 10.3389/fmed.2026.1877209 (PMC13585711; doi:10.3389/fmed.2026.1877209)
Supplement: Supplementary file 2 [file Supplementary_file_2.doc]

S3 Supplemental Table 2: Side Effects During Induction Period Between Two Groups.

|  | Group O (n,%) |  | Group N (n,%) | *p-value* |
| --- | --- | --- | --- | --- |
| Hypotension | 10(33.3%) |  | 7(23.3%) | 0.390 |
| Bradycardia | 1(3.3%) |  | 1(3.3%) | > 0.99 |
| Respiratory depression | 8(26.7%) |  | 8(26.7%) | > 0.99 |
| Hiccup | 2(6.6%) |  | 1(6.6%) | 0.554 |

Notes: Data shown as number (%), *p* < 0.05 was deemed statistically significant.
