# Supplementary material for: An evaluation of the relationship between BIS and MOAA/S during remimazolam-sufentanil induction: a prospective observational study
Source: Front Med (Lausanne). 2026 Sep 4;13:1877209. doi: 10.3389/fmed.2026.1877209 (PMC13585711; doi:10.3389/fmed.2026.1877209)
Supplement: Supplementary file 3 [file Supplementary_file_3.doc]

S3 Supplemental Table 3: The comparison results of sedation monitoring indicators and the results of the control study.

|  | Accounting-based measures | Research results | Age(y) | Drug | Statistical Analysis |
| --- | --- | --- | --- | --- | --- |
| Lim S 2022^[12]^ | MOAA/S, PLE, BIS | Mass energy equivalence principle | 20-80 | Dexmedetomidine | Spearman's correlation coefficient and Prediction probability (Pk) values |
| Shajahan MS 2023^[13]^ | PSI, BIS, MOAA/S | Mass energy equivalence principle | 18-65 | propofol | analysis of variance |
| Avci S 2019^[31]^ | BIS and RSS scores | high correlation | >18 | Midazolam propofol | The Pearson correlation analysis |
| Sachdeva A 2023^[32]^ | BIS and RSS scores | Strong correlation | >65 | Midazolam propofol | The chi-square/Fisher's exact test |
| Bae MI 2024^[8]^ | BIS and PSI | Acceptable correlation | 18-60 | remimazolam | Spearman's correlation coefficient and Prediction probability (Pk) values |
| Zhao TY 2023^[9]^ | BIS and PSI | strongly associated | 65-80 | remimazolam | Pearson’s Correlation Coefficient (r) |
| Junli Zheng et al(This study) | BIS and MOAA/S | Poor consistence | >18 | remimazolam | a consistency test (Kappa) |

Note: BIS: Bispectral Index; RSS: Ramsay Sedation Scale; MOAA/S: modified observer's assessment of alertness/sedation; PLE: phase lag entropy; PSI: The patient state index;
